# Combining Non-Pharmacological Treatments with Pharmacotherapies for Neurological Disorders: A Unique Interface of the Brain, Drug–Device, and Intellectual Property

**DOI:** 10.3389/fneur.2014.00126

**Published:** 2014-07-14

**Authors:** Grzegorz Bulaj

**Affiliations:** ^1^Department of Medicinal Chemistry, Skaggs Pharmacy Institute, College of Pharmacy, University of Utah, Salt Lake City, UT, USA

**Keywords:** chronic disease, psychiatric disorders, dopamine, Mozart, epilepsy, depression, fatigue

## Abstract

Mobile medical applications (mHealth), music, and video games are being developed and tested for their ability to improve pharmacotherapy outcomes and medication adherence. Pleiotropic mechanism of music and gamification engages an intrinsic motivation and the brain reward system, supporting therapies in patients with neurological disorders, including neuropathic pain, depression, anxiety, or neurodegenerative disorders. Based on accumulating results from clinical trials, an innovative combination treatment of epilepsy seizures, comorbidities, and the medication non-adherence can be designed, consisting of antiepileptic drugs and disease self-management software delivering clinically beneficial music. Since creative elements and art expressed in games, music, and software are copyrighted, therefore clinical and regulatory challenges in developing copyrighted, drug–device therapies may be offset by a value proposition of the exclusivity due to the patent–independent protection, which can last for over 70 years. Taken together, development of copyrighted non-pharmacological treatments (e-therapies), and their combinations with pharmacotherapies, offer incentives to chronically ill patients and outcome-driven health care industries.

## Introduction

Mobile medical applications (mHealth apps) emerge as daily companions to help patients, clinicians, and pharmacists (1–10). There is a growing number of self-management applications including those for chronic pain (11, 12), asthma (13), or mental health (14, 15). One example of an FDA-cleared mobile therapy is BlueStar^®^, the clinical and behavioral self-management platform for patients with type 2 diabetes, which was shown to improve control of glucose blood levels (16–18). Mobile medical applications cleared by the FDA range from diagnostic and monitoring platforms to a sound therapy or interactive medication reminders (19). The FDA intends to exercise enforcement discretion for mobile medical applications, which aim to help patients with disease self-management or medication adherence, while those intended to perform medical device functions, may require approval or clearance (2). The exponential growth of mobile medical applications has resulted in a lag of reports from larger scale, randomized controlled trials (RCTs) to support their clinical utilities (3, 20).

Interactive technologies targeting healthy behaviors and therapy outcomes include video games (21). Clinical utility of serious video games (e-therapies or therapeutic games) has been studied in patients with various chronic conditions including depression, Parkinson’s disease, asthma, diabetes, cancer, or stroke (22–26). Table S1 in Supplementary Material provides examples of mobile apps and games for the treatment of depression, anxiety, dementia, pain, attention deficit hyperactivity disorder (ADHD), and cerebral palsy. One example of a serious video game is “Re-Mission,” a game specifically designed for cancer patients and shown to improve behavioral outcomes, cancer-related knowledge, and medication adherence during chemotherapy in adolescent patients with acute leukemia, lymphoma, and soft-tissue sarcoma (27, 28). In RCTs, playing video games was found to be clinically effective in treating depression (25, 29). The use of video games to modulate the brain neuroplasticity also improved age-related neuronal deficits and enhanced cognitive functions in older adults (30). Video games are explored as a preventive medicine strategy against HIV infections (31), or obesity (32). Fun and game-based principles (gamification) in serious video games are important elements when targeting intrinsic motivation to improve health behaviors (21). In 2014, the FDA cleared a motion-capture video game, developed by Jintronix as a rehabilitation system for stoke patients, or those with traumatic brain injury.

Music has been applied as a non-pharmacological treatment mainly for neurological conditions. The pleiotropic nature of music is mediated by neurochemical changes in the brain, endocrinological, and immune systems (33–35). A therapeutic potential of music to treat psychiatric disorders was recently reviewed (33). Recent studies show clinical applications of music for epilepsy patients (36, 37), including listening to the Mozart K.448 sonata, which was shown to reduce frequency of epileptiform discharges (38–43), and to reduce seizure frequencies (40, 43, 44). Music-supported therapies include indications such as pain (45–48), stroke (49, 50), dementia (51, 52), depression (53), or anxiety (54, 55). A therapeutic music video intervention was shown to improve resilience in cancer patients being treated with hematopoietic stem cells (56). Given popularity and convenience of listening to music, this non-pharmacological treatment can be easily incorporated into mobile medical applications.

**Figure 1:**
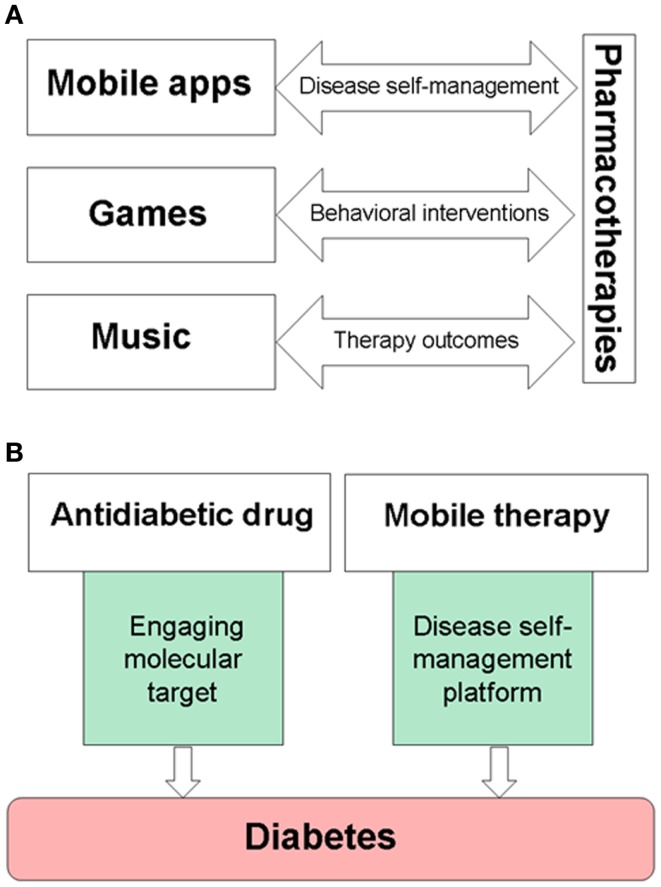
**Examples of drug–device interfaces created by mobile medical applications, serious video games, or music**. **(A)** Electronic devices are used to provide music, games, and mobile medical applications, while also becoming delivery systems for disease self-management platforms. **(B)** An example of combining pharmacotherapies with medical devices for the treatment of diabetes. Mobile therapies, such as BlueStar^®^, can support disease-specific, healthy behaviors of a patient, and remind about taking prescribed medications on-time.

Over the last decade, it has been recognized that a significant number of patients with chronic diseases fail to take medications as prescribed (57, 58). Medication non-adherence is considered as a serious global health care problem (59). While clinical aspects of medication adherence are studied, economic costs of medication non-adherence are estimated as $100–290 billion per year in additional medical spending paid by the US health care (57) and $564 billion in lost revenues of the global pharmaceutical industry (60). Current strategies to improve medication adherence include less frequent dosing, extended-release formulations, novel drug delivery devices, providing external incentives, or support-based interventions. Medication non-adherence is a patient behavioral aspect associated with all chronic diseases. Health care industry is embracing gamification and electronic platforms in patient care (61). Clinical studies of mobile medical applications, serious video games, or music show their promise as tools to improve therapy outcomes, disease self-management, or medication adherence for chronically ill patients (Figure 1A). This article discusses opportunities for creating and clinical development of non-pharmacological treatments, which can be integrated into specific combination therapies for chronic diseases.

## Integrating Gamification and Music into Therapies – Dopaminergic Connections

FDA-cleared mobile and video game therapies, such as BlueStar^®^ and Jintronix Rehabilitation System, show new opportunities to integrate drugs with devices, thereby bridging pharmacotherapies with disease self-management. Figure 1B shows an example of pharmacotherapy in combination with a mobile platform for the treatment of diabetes. Since studies encourage clinical use of music for pain relief (48, 62, 63), parallel needs for more personalized treatments of neuropathic pain and for improving pain relief (64) can be addressed by a combination of an appropriate analgesic and pain self-management platforms with music and games for pain relief. Therapeutic interactive voice response has been shown to reduce use of opioids and non-steroidal anti-inflammatory drugs (NSAIDs) (65). Cancer patients may benefit from combining chemotherapy with a serious video game, such as “Re-Mission” (27, 28) or the Patient Empowerment Exercise Video Game (22) which target comorbidities including depression, anxiety, or fatigue. Gamification is a tool to engage intrinsic motivation using such features as choice and decision-making, levels and challenge, fantasy and curiosity (66). Therapeutic video games and self-acquired rewards engage the brain reward system in active players (67, 68), therefore integrating gamification into self-management platforms is also useful for motivating and rewarding patients while learning and reinforcing disease-specific healthy habits.

Studies on physiological effects of music and games begin to indicate multiple effects on the nervous and endocrine systems, including music-induced modulation of various neurotransmitters and hormones (34, 35). An overlapping mechanism of gaming and music includes activation of the mesolimbic system and dopaminergic neurotransmission in the brain (67–72). Game- or music-evoked dopamine release involving dopamine D2 receptors was shown by positron emission tomography using 11C-labeled raclopride (69, 71). Table S2 in Supplementary Material provides examples of functional magnetic resonance imaging (fMRI) studies aimed to elucidate music or games mechanism of actions. Both gaming and music activate nucleus accumbens in the mesolimbic reward circuitry, as well as parts of prefrontal cortex (67, 68, 72, 73). Mesolimbic dopamine plays important roles in learning and motivation (74, 75), thus activation of the dopaminergic signaling by therapeutic games or/and tailored music can facilitate behavioral and pharmacological interventions in patients. Targeting the dopamine system by playing video games improved symptoms in schizophrenia patients (76), whereas clinical studies of games for depression (25) or Parkinson’s disease (24, 77), encourage similar strategies for migraines or addiction. Taken together, the dopaminergic mechanisms of music and games offer opportunities for coupling the pleasurable brain reward system with intrinsic motivation and the formation of healthy habits, resulting in unique disease self-management platforms. Given pleiotropic effects of music and games, mechanism-based combinations of these non-pharmacological treatments with specific drugs can improve therapy outcomes for various neurological diseases.

## Redefining a Combination Therapy for Epilepsy

**Figure 2:**
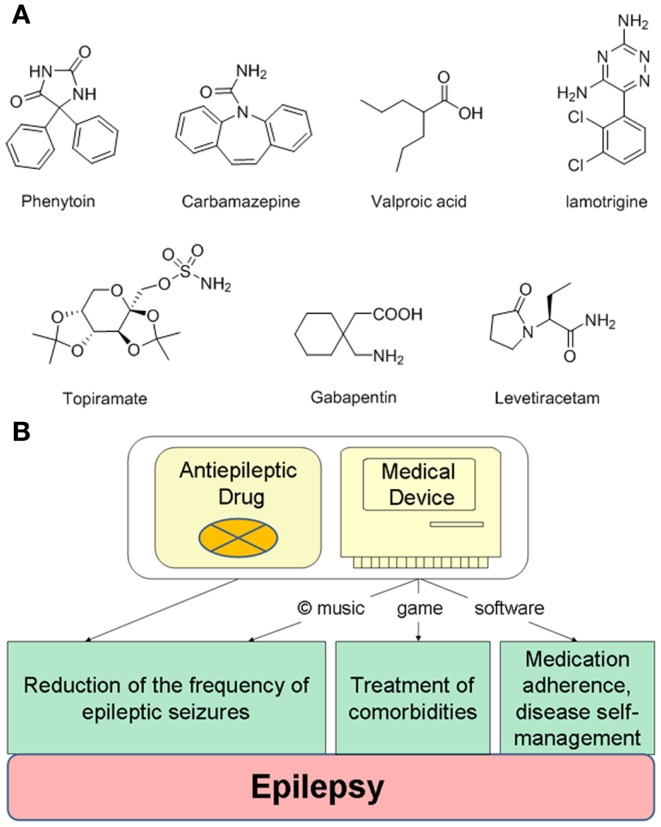
**Integrating antiepileptic drugs into a copyrighted combination therapy for the treatment of epilepsy**. **(A)** Examples of generic antiepileptic drugs. **(B)** Designing an integrated combination therapy consisting of antiepileptic drugs and copyrighted non-pharmacological components delivered by an electronic device. While antiepileptic drugs and music can treat epileptic seizures, a self-management digital platform can include specifically designed games for the treatment of depression or anxiety.

Designing innovative drug–device combination therapies can be illustrated using the treatment of epilepsy. Managing epilepsy is challenging due to: (i) significant resistance to antiepileptic drugs (AEDs), (ii) compromised medication adherence, and (iii) significant prevalence of comorbidities, such as anxiety or depression (78). Despite many AEDs approved for the treatment of epilepsy (Figure 2A), estimated 25–40% of patients with epilepsy are refractory to current AEDs (79). An approximate 50–60% medication adherence rate among epilepsy patients results in increased risks of seizures and mortality (80–82), while the main comorbidity, depression, is also difficult to treat with antidepressant drugs (83). To help epilepsy patients with disease self-management, currently available mobile devices include a SmartWatch, which is capable of detecting movements associated with seizures, as well as mobile applications helping to self-report and record seizures, manage medications, or learn more facts about epilepsy.

Accumulating clinical evidence suggests that listening to the Mozart’s K.448 results in reduction of generalized and focal seizures in epilepsy patients (36, 37). The K.448 was also effective in reducing seizure frequencies in children with pharmacoresistant epilepsy (84), and in patients following their first unprovoked seizures (43). Using simultaneous electroencephalogram and electrocardiogram recordings, the reduction of epileptiform discharges by K.448 and K.545 was shown to be accompanied by activation of the parasympathetic system (42). While an anticonvulsant mechanism of action for audiogenic stimulation remains unknown, reports also suggest a role of mirror neurons or/and neurotransmitters pathways including the dopaminergic system (36, 37). Genetic, pharmacological, and imaging studies support the dopamine signaling in epilepsy and epileptogenesis, including D2 receptors (85–88).

To improve pharmacotherapy, manage comorbidities and medication adherence for epilepsy patients, the drug–device combination therapy can include a specific AED and a matching medical device delivering non-pharmacological elements (Figure 2B). The most apparent non-pharmacological component could be music, which reduces seizures [such as the Mozart’s K.448 or K.545 (41)]. To treat symptoms of depression or anxiety, often experienced by epilepsy patients, the self-management platform can include a game comprising cognitive behavioral therapy elements (29), or even a combination of music and games as musical games. Such platforms may also help manage seizure-precipitating stress by modulating cortical responses (89). Noteworthy, a long-term stimulation of nucleus accumbens can decrease seizure severity (90), while integrating music into the treatments of epilepsy comorbidities was recently emphasized (91). Additional non-pharmacological components delivered by the medical device can include medication adherence reminders and other features to self-report and track seizures. This integrated treatment may appeal to pediatric epilepsy patients, since non-adherence to AEDs within the first 6 months significantly affects long-term rates of becoming seizure-free (92).

Creating therapeutic music to reduce epileptic seizures extends to translational research, since music can be subject to preclinical screening [for example, in such indications as addiction (93)]. In animal testing, exposure to the Mozart K.448 resulted in: (i) reduction of spontaneous absence seizure and high-voltage rhythmic spike discharges in Long Evans rats (94), (ii) increased dopamine levels in rat brains (95), and (iii) decreased corticosterone (95, 96). Positive effects of the K.448 music on spatial–temporal learning were reported for rats and mice (97–99). Since clinical data suggest that K.448 can reduce seizures in patients with refractory epilepsy (84), studies on interactions between K.448 and AEDs in animal models of epilepsy may help to elucidate the mechanism of action of K.448 while increasing the potency of the AEDs. Comparing preclinical and clinical effects of music in other medical indications, such as depression, anxiety, addiction, or neurodegenerative disorders may validate the translational research for using specific auditory stimulations with drugs.

## Copyrights as Incentives for Creating Non-Pharmacological Treatments

An incentive for creating and clinical development of non-pharmacological therapies can be their copyrighted works, which are protected by the intellectual property (IP) law. Unlike patents protecting ideas, copyright protects the expression of an idea found in creative elements and art found in digital media, software, electronic games, and music. In the US, the Copyright Act of 1976 offers the benefit a long-term protection lasting not less than 70 years, and up to 120 years. Copyright protection is automatic as soon as it is created, although a copyright registration offers legal advantages in case of any future infringements. Furthermore, copyright is recognized and protected in most countries under national and international law through the Universal Copyright Convention and the Berne Convention which the US joined as a member in 1955 and 1989, respectively.

In a web-based era, developing games or music intended for clinical use create opportunities for “off-label” uses, whereas classical music which exists in public domain (such as Mozart’s K.448 or K.545 sonatas) presents the exclusivity challenge. Re-recording of specific pieces of classical music may offer recording copyrights, while re-composing and modifying individual pieces of public-domain music intended for clinical development may offer an additional level of protection before evaluating therapeutic claims. The copyright protection for the music created, re-composed, and recorded “for hire” can last 120 years. Clinically validated music can be used as a “stand-alone” non-pharmacological therapy, or can be integrated into disease self-management digital platforms or therapeutic games (Figure 2B). The Digital Millenium Copyright Act from 1998 is the law protecting copyrighted art and works used in digital media against infringement and piracy. In contrast to music, electronic and video games pose complex copyright challenges due to multiple components of art involved in digital technologies (100).

## Opportunities and Challenges of the Copyrighted Combination Therapies

Using mobile applications, playing electronic games, or listening to music are daily practices of many patients taking medications. As mentioned earlier, games and music target intrinsic motivation and the reward system, becoming useful “personalized medicine” tools to improve patient compliance with their prescribed pharmacotherapies. For chronically ill patients at risk for medication non-adherence, gamification strategies are alternatives to providing external incentives to reinforce taking drugs as prescribed, given that patient motivation may decrease once incentives are discontinued (101). Combination of pharmacotherapy and gamification can improve treatments for chronic diseases with inflammatory components (such as rheumatoid arthritis), which can be lessen by behavioral changes and self-management platforms (102). Given advances in genomics, individuals carrying mutations in susceptibility genes for chronic diseases may benefit from non-pharmacological and behavioral interventions. In addition to encouraging development of preventive medicine strategies, copyrights can also provide incentives to create innovative treatments for rare genetic disorders, for example epileptic encephalopathies.

Since medication non-adherence affects approximately half of the prescribed therapeutics for chronic disorders, drug discovery and clinical development efforts will benefit from applications of the copyrighted combination therapies due to integration of pharmacotherapy outcomes and medication adherence. This aspect becomes important when translating discoveries of new molecular targets into combination therapies: for example, the treatment of epilepsy emphasize needs for network-based polypharmacies (103). Copyrighted therapies may also encourage clinical development of new combination therapies with clinically beneficial compounds existing in public domain (e.g., curcumin), due to the IP exclusivity under the premarket approval (PMA) application. Copyrighted combination products may appeal to developing therapies for neurodegenerative diseases. For example, having 70 years protection of the copyrighted combination therapy for the Alzheimer disease would facilitate balancing decisions between clinical and business objectives related to lengthy clinical trials. Pharmaceutical industry is recognizing both opportunities and challenges in applying mobile medical applications and gamification in patient care (104, 105). Since changing patent guidelines can affect drug discovery efforts (106), copyrights can provide incentives to transform generic drugs into innovative treatments of chronic diseases with improve medication adherence and outcomes.

Herein, it is prudent to emphasize challenging times for the regulatory agencies and for those who seek approval/clearance of mobile medical applications and their combinations with pharmacotherapies (1, 107). Integrating drugs with devices is commonly associated with combination products such as pen injectors and insulin, transdermal patches, drug-eluting stents, or drug creams with light sources (108, 109). The long-term prospects of the copyrighted combination therapies include becoming the combination products. For example, given advancements in medical technologies at the interface of drugs and devices, the FDA established the Office of Combination Products (110, 111). Efforts to reach PMA applications for the copyrighted combination products may be incentivized by the long-term exclusivity of specific medical treatment due to the copyright protection. Creation, translational research, and clinical validation of copyrighted non-pharmacological treatments is a long and challenging frontier, requiring cross-technological interactions among pharmaceutical-biotech companies, electronic and mobile devices, and those providing the creative works such as software, video games, or music.

Clinical development of the non-pharmacological therapies like therapeutic games or music, and their combinations with the pharmacotherapies, carry inherent risks related to safety (adverse effects) and efficacy (tolerance, addiction). Developing tolerance to non-pharmacological treatments is unknown, whereas addictive properties of gaming can be addressed by appropriate design features. Current understanding of mechanisms of musicogenic seizures and photosensitive epilepsy can facilitate designing music and games to avoid proconvulsant properties. Incorporation of features such as patient preference to have choices in selecting music for the therapy is offset by unknown variables in response to music due to differences in a patient genetics and personality [for example individual responses to music for pain treatment were reported (63)]. Large-scale RCTs will test therapeutic utility of non-pharmacological treatments and their combinations with drugs, and will advance clinical knowledge and design of such innovative treatments for neurological and other chronic medical conditions.

## Conclusion

Taken together, creating and developing copyrighted music, games, and medical software to be combined with pharmacotherapies provides an opportunity to deliver novel treatments for patients with neurological and other chronic diseases. Neuroscience-centered, innovative drug–device platforms can facilitate delivery of more personalized and integrated treatments of the target disease and associated comorbidities, resulting in improved therapy outcomes and medication adherence. Mechanism-based combinations of pharmacotherapies with non-pharmacological treatments offer win–win solutions for all health care stake-holders.

## Conflict of Interest Statement

Grzegorz Bulaj is a co-inventor of the patent “Disease Therapy Game Technology,” owned by the University of Utah. Grzegorz Bulaj is a scientific cofounder of NeuroAdjuvants, Inc., a company focused on developing peptide-based drugs that cross the blood–brain barrier for the treatments of epilepsy and pain.

## Supplementary Material

The Supplementary Material for this article can be found online at http://www.frontiersin.org/Journal/10.3389/fneur.2014.00126/abstract

## Abbreviations

AEDs, antiepileptic drugs; FDA, Food and Drug Administration; IP, intellectual property; NSAIDS, non-steroidal anti-inflammatory drugs; PMA, premarket approval; RCTs, randomized controlled trials.

## References

[1] Charani E Castro-Sanchez E Moore LS Holmes A Do smartphone applications in healthcare require a governance and legal framework? It depends on the application! BMC Med (2014) 12:29 10.1186/1741-7015-12-29 24524344 PMC3929845

[2] Food and Drug Administration. Mobile Medical Applications: Guidance for Industry and Food and Drug Administration Staff (2013). Available from: http://www.fda.gov/downloads/MedicalDevices/./UCM263366.pdf

[3] de Jongh T Gurol-Urganci I Vodopivec-Jamsek V Car J Atun R Mobile phone messaging for facilitating self-management of long-term illnesses. Cochrane Database Syst Rev (2013) 12:CD007459 10.1002/14651858.CD007459.pub2 23235644 PMC6486189

[4] Jones KR Lekhak N Kaewluang N Using mobile phones and short message service to deliver self-management interventions for chronic conditions: a meta-review. Worldviews Evid Based Nurs (2014) 11:81–8 10.1111/wvn.12030 24597522

[5] Hayes DF Markus HS Leslie RD Topol EJ Personalized medicine: risk prediction, targeted therapies and mobile health technology. BMC Med (2014) 12:37 10.1186/1741-7015-12-37 24580858 PMC3938085

[6] Finitsis DJ Pellowski JA Johnson BT Text message intervention designs to promote adherence to antiretroviral therapy (ART): a meta-analysis of randomized controlled trials. PLoS One (2014) 9:e88166 10.1371/journal.pone.0088166 24505411 PMC3914915

[7] Free C Phillips G Galli L Watson L Felix L Edwards P The effectiveness of mobile-health technology-based health behaviour change or disease management interventions for health care consumers: a systematic review. PLoS Med (2013) 10:e1001362 10.1371/journal.pmed.1001362 23349621 PMC3548655

[8] Aikens JE Zivin K Trivedi R Piette JD Diabetes self-management support using mHealth and enhanced informal caregiving. J Diabetes Complications (2014) 28:171–6 10.1016/j.jdiacomp.2013.11.008 24374137 PMC3943823

[9] Dayer L Heldenbrand S Anderson P Gubbins PO Martin BC Smartphone medication adherence apps: potential benefits to patients and providers. J Am Pharm Assoc (2003) 53:172–81 10.1331/JAPhA.2013.12202 23571625 PMC3919626

[10] Vervloet M van Dijk L Santen-Reestman J van Vlijmen B van Wingerden P Bouvy ML SMS reminders improve adherence to oral medication in type 2 diabetes patients who are real time electronically monitored. Int J Med Inform (2012) 81:594–604 10.1016/j.ijmedinf.2012.05.005 22652012

[11] Reynoldson C Stones C Allsop M Gardner P Bennett MI Closs SJ Assessing the quality and usability of smartphone apps for pain self-management. Pain Med (2014) 15(6):898–909 10.1111/pme.12327 24422990

[12] Wallace LS Dhingra LK A systematic review of smartphone applications for chronic pain available for download in the United States. J Opioid Manag (2014) 10:63–8 10.5055/jom.2014.0193 24604571

[13] Marcano Belisario JS Huckvale K Greenfield G Car J Gunn LH Smartphone and tablet self management apps for asthma. Cochrane Database Syst Rev (2013) 11:CD010013 10.1002/14651858.CD010013.pub2 24282112 PMC6486323

[14] Andrews G Cuijpers P Craske MG McEvoy P Titov N Computer therapy for the anxiety and depressive disorders is effective, acceptable and practical health care: a meta-analysis. PLoS One (2010) 5:e13196 10.1371/journal.pone.0013196 20967242 PMC2954140

[15] Donker T Petrie K Proudfoot J Clarke J Birch MR Christensen H Smartphones for smarter delivery of mental health programs: a systematic review. J Med Internet Res (2013) 15:e247 10.2196/jmir.2791 24240579 PMC3841358

[16] Quinn CC Clough SS Minor JM Lender D Okafor MC Gruber-Baldini A WellDoc mobile diabetes management randomized controlled trial: change in clinical and behavioral outcomes and patient and physician satisfaction. Diabetes Technol Ther (2008) 10:160–8 10.1089/dia.2008.0283 18473689

[17] Quinn CC Gruber-Baldini AL Shardell M Weed K Clough SS Peeples M Mobile diabetes intervention study: testing a personalized treatment/behavioral communication intervention for blood glucose control. Contemp Clin Trials (2009) 30:334–46 10.1016/j.cct.2009.02.004 19250979

[18] Quinn CC Shardell MD Terrin ML Barr EA Ballew SH Gruber-Baldini AL Cluster-randomized trial of a mobile phone personalized behavioral intervention for blood glucose control. Diabetes Care (2011) 34:1934–42 10.2337/dc11-0366 21788632 PMC3161305

[19] Shuren J The FDA’s role in the development of medical mobile applications. Clin Pharmacol Ther (2014) 95:485–8 10.1038/clpt.2014.45 24747239

[20] Hieftje K Edelman EJ Camenga DR Fiellin LE Electronic media-based health interventions promoting behavior change in youth: a systematic review. JAMA Pediatr (2013) 167:574–80 10.1001/jamapediatrics.2013.1095 23568703 PMC3733329

[21] Baranowski T Buday R Thompson D Lyons EJ Lu AS Baranowski J Developing games for health behavior change: getting started. Games Health J (2013) 2:183–90 10.1089/g4h.2013.0048 24443708 PMC3892986

[22] Bruggers CS Altizer RA Kessler RR Caldwell CB Coppersmith K Warner L Patient-empowerment interactive technologies. Sci Transl Med (2012) 4:152s116 10.1126/scitranslmed.3004009 22993292

[23] Lieberman DA Video games for diabetes self-management: examples and design strategies. J Diabetes Sci Technol (2012) 6:802–6 10.1177/193229681200600410 22920805 PMC3440150

[24] Esculier JF Vaudrin J Beriault P Gagnon K Tremblay LE Home-based balance training programme using Wii fit with balance board for Parkinsons’s disease: a pilot study. J Rehabil Med (2012) 44:144–50 10.2340/16501977-0922 22266676

[25] Russoniello CV Fish M O’Brien K The efficacy of casual videogame play in reducing clinical depression: a randomized controlled study. Games Health J (2013) 2:341–6 10.1089/g4h.2013.0010 26197075

[26] Kottink AIR Prange GB Krabben T Rietman JS Buurke JH Gaming and conventional exercise for improvement of arm function after stroke: a randomized controlled pilot study. Games Health J (2014) 3:184–91 10.1089/g4h.2014.0026 26196178

[27] Beale IL Kato PM Marin-Bowling VM Guthrie N Cole SW Improvement in cancer-related knowledge following use of a psychoeducational video game for adolescents and young adults with cancer. J Adolesc Health (2007) 41:263–70 10.1016/j.jadohealth.2007.04.006 17707296

[28] Kato PM Cole SW Bradlyn AS Pollock BH A video game improves behavioral outcomes in adolescents and young adults with cancer: a randomized trial. Pediatrics (2008) 122:e305–17 10.1542/peds.2007-3134 18676516

[29] Merry SN Stasiak K Shepherd M Frampton C Fleming T Lucassen MF The effectiveness of SPARX, a computerised self help intervention for adolescents seeking help for depression: randomised controlled non-inferiority trial. BMJ (2012) 344:e2598 10.1136/bmj.e2598 22517917 PMC3330131

[30] Anguera JA Boccanfuso J Rintoul JL Al-Hashimi O Faraji F Janowich J Video game training enhances cognitive control in older adults. Nature (2013) 501:97–101 10.1038/nature12486 24005416 PMC3983066

[31] Hieftje K Rosenthal MS Camenga DR Edelman EJ Fiellin LE A qualitative study to inform the development of a video game for adolescent HIV prevention. Games Health J (2012) 1:294–8 10.1089/g4h.2012.0025 24078897 PMC3782854

[32] Simons M Chinapaw MJ van de Bovenkamp M de Boer MR Seidell JC Brug J Active video games as a tool to prevent excessive weight gain in adolescents: rationale, design and methods of a randomized controlled trial. BMC Public Health (2014) 14:275 10.1186/1471-2458-14-275 24661535 PMC3987926

[33] Koelsch S Brain correlates of music-evoked emotions. Nat Rev Neurosci (2014) 15:170–80 10.1038/nrn3666 24552785

[34] Chanda ML Levitin DJ The neurochemistry of music. Trends Cogn Sci (2013) 17(4):179–93 10.1016/j.tics.2013.02.007 23541122

[35] Fancourt D Ockelford A Belai A The psychoneuroimmunological effects of music: a systematic review and a new model. Brain Behav Immun (2014) 36:15–26 10.1016/j.bbi.2013.10.014 24157429

[36] Dastgheib SS Layegh P Sadeghi R Foroughipur M Shoeibi A Gorji A The effects of Mozart’s music on interictal activity in epileptic patients: systematic review and meta-analysis of the literature. Curr Neurol Neurosci Rep (2014) 14:420 10.1007/s11910-013-0420-x 24272274

[37] Lin L Yang R Using music to treat epilepsy in children: a review. Music Med (2013) 5:242–7 10.1177/194386211350050618627794

[38] Hughes JR Daaboul Y Fino JJ Shaw GL The “Mozart effect” on epileptiform activity. Clin Electroencephalogr (1998) 29:109–19 10.1177/155005949802900301 9660010

[39] Lin LC Lee WT Wu HC Tsai CL Wei RC Jong YJ Mozart K.448 and epileptiform discharges: effect of ratio of lower to higher harmonics. Epilepsy Res (2010) 89:238–45 10.1016/j.eplepsyres.2010.01.007 20129759

[40] Lin LC Lee WT Wu HC Tsai CL Wei RC Mok HK The long-term effect of listening to Mozart K.448 decreases epileptiform discharges in children with epilepsy. Epilepsy Behav (2011) 21:420–4 10.1016/j.yebeh.2011.05.015 21689988

[41] Lin LC Lee MW Wei RC Mok HK Wu HC Tsai CL Mozart k.545 mimics Mozart k.448 in reducing epileptiform discharges in epileptic children. Evid Based Complement Alternat Med (2012) 2012:607517 10.1155/2012/607517 23304207 PMC3523174

[42] Lin LC Chiang CT Lee MW Mok HK Yang YH Wu HC Parasympathetic activation is involved in reducing epileptiform discharges when listening to Mozart music. Clin Neurophysiol (2013) 124:1528–35 10.1016/j.clinph.2013.02.021 23540417

[43] Lin LC Lee MW Wei RC Mok HK Yang RC Mozart K.448 listening decreased seizure recurrence and epileptiform discharges in children with first unprovoked seizures: a randomized controlled study. BMC Complement Altern Med (2014) 14:17 10.1186/1472-6882-14-17 24410973 PMC3893543

[44] Bodner M Turner RP Schwacke J Bowers C Norment C Reduction of seizure occurrence from exposure to auditory stimulation in individuals with neurological handicaps: a randomized controlled trial. PLoS One (2012) 7:e45303 10.1371/journal.pone.0045303 23071510 PMC3469625

[45] Bernatzky G Presch M Anderson M Panksepp J Emotional foundations of music as a non-pharmacological pain management tool in modern medicine. Neurosci Biobehav Rev (2011) 35:1989–99 10.1016/j.neubiorev.2011.06.005 21704068

[46] Cole LC Lobiondo-Wood G Music as an adjuvant therapy in control of pain and symptoms in hospitalized adults: a systematic review. Pain Manag Nurs (2014) 15:406–25 10.1016/j.pmn.2012.08.010 23107431

[47] Guetin S Ginies P Siou DK Picot MC Pommie C Guldner E The effects of music intervention in the management of chronic pain: a single-blind, randomized, controlled trial. Clin J Pain (2012) 28:329–37 10.1097/AJP.0b013e31822be973 22001666

[48] Garza-Villarreal EA Wilson AD Vase L Brattico E Barrios FA Jensen TS Music reduces pain and increases functional mobility in fibromyalgia. Front Psychol (2014) 5:90 10.3389/fpsyg.2014.00090 24575066 PMC3920463

[49] Rodriguez-Fornells A Rojo N Amengual JL Ripolles P Altenmuller E Munte TF The involvement of audio-motor coupling in the music-supported therapy applied to stroke patients. Ann N Y Acad Sci (2012) 1252:282–93 10.1111/j.1749-6632.2011.06425.x 22524370

[50] Altenmuller E Schlaug G Neurobiological aspects of neurologic music therapy. Music Med (2013) 5:210–6 10.1177/1943862113505328

[51] Ueda T Suzukamo Y Sato M Izumi S Effects of music therapy on behavioral and psychological symptoms of dementia: a systematic review and meta-analysis. Ageing Res Rev (2013) 12:628–41 10.1016/j.arr.2013.02.003 23511664

[52] Vasionyte I Madison G Musical intervention for patients with dementia: a meta-analysis. J Clin Nurs (2013) 22:1203–16 10.1111/jocn.12166 23574287

[53] Erkkila J Punkanen M Fachner J Ala-Ruona E Pontio I Tervaniemi M Individual music therapy for depression: randomised controlled trial. Br J Psychiatry (2011) 199:132–9 10.1192/bjp.bp.110.085431 21474494

[54] Bradt J Dileo C Shim M Music interventions for preoperative anxiety. Cochrane Database Syst Rev (2013) 6:CD006908 10.1002/14651858.CD006908.pub2 23740695 PMC9758540

[55] Chlan LL Weinert CR Heiderscheit A Tracy MF Skaar DJ Guttormson JL Effects of patient-directed music intervention on anxiety and sedative exposure in critically ill patients receiving mechanical ventilatory support: a randomized clinical trial. JAMA (2013) 309:2335–44 10.1001/jama.2013.5670 23689789 PMC3683448

[56] Robb SL Burns DS Stegenga KA Haut PR Monahan PO Meza J Randomized clinical trial of therapeutic music video intervention for resilience outcomes in adolescents/young adults undergoing hematopoietic stem cell transplant: a report from the children’s oncology group. Cancer (2014) 120:909–17 10.1002/cncr.28355 24469862 PMC3947727

[57] Osterberg L Blaschke T Adherence to medication. N Engl J Med (2005) 353:487–97 10.1056/NEJMra050100 16079372

[58] Brown MT Bussell JK Medication adherence: WHO cares? Mayo Clin Proc (2011) 86:304–14 10.4065/mcp.2010.0575 21389250 PMC3068890

[59] World Health Organization. Adherence to Long Term Therapies: Evidence for Action. Geneva: World Health Organization (2003).

[60] Forissier T Firlik K Estimated Annual Pharmaceutical Revenue Loss Due to Medication Non-Adherence. Paris: Capgemini Consulting (2012). p. 1–20

[61] Landwehr B Big games: one company’s experience with gamification of health. Games Health J (2014) 3:64–6 10.1089/g4h.2014.0007 26196045

[62] Bradshaw DH Chapman CR Jacobson RC Donaldson GW Effects of music engagement on responses to painful stimulation. Clin J Pain (2012) 28:418–27 10.1097/AJP.0b013e318236c8ca 22395335 PMC3348972

[63] Bradshaw DH Donaldson GW Jacobson RC Nakamura Y Chapman CR Individual differences in the effects of music engagement on responses to painful stimulation. J Pain (2011) 12:1262–73 10.1016/j.jpain.2011.08.010 22071366 PMC3258530

[64] Chaparro LE Wiffen PJ Moore RA Gilron I Combination pharmacotherapy for the treatment of neuropathic pain in adults. Cochrane Database Syst Rev (2012) 7:CD008943 10.1002/14651858.CD008943.pub2 22786518 PMC6481651

[65] Naylor MR Naud S Keefe FJ Helzer JE Therapeutic interactive voice response (TIVR) to reduce analgesic medication use for chronic pain management. J Pain (2010) 11:1410–9 10.1016/j.jpain.2010.03.019 20620119 PMC3045626

[66] Moon H Baek Y Exploring variables affecting player’s intrinsic motivation in educational games. The 17th International Conference on Computers in Education Hong Kong: Asia-Pacific Society for Computers in Education (2009).

[67] Cole SW Yoo DJ Knutson B Interactivity and reward-related neural activation during a serious videogame. PLoS One (2012) 7:e33909 10.1371/journal.pone.0033909 22442733 PMC3307771

[68] Katsyri J Hari R Ravaja N Nummenmaa L Just watching the game ain’t enough: striatal fMRI reward responses to successes and failures in a video game during active and vicarious playing. Front Hum Neurosci (2013) 7:278 10.3389/fnhum.2013.00278 23781195 PMC3680713

[69] Koepp MJ Gunn RN Lawrence AD Cunningham VJ Dagher A Jones T Evidence for striatal dopamine release during a video game. Nature (1998) 393:266–8 10.1038/30498 9607763

[70] Gold BP Frank MJ Bogert B Brattico E Pleasurable music affects reinforcement learning according to the listener. Front Psychol (2013) 4:541 10.3389/fpsyg.2013.00541 23970875 PMC3748532

[71] Salimpoor VN Benovoy M Larcher K Dagher A Zatorre RJ Anatomically distinct dopamine release during anticipation and experience of peak emotion to music. Nat Neurosci (2011) 14:257–62 10.1038/nn.2726 21217764

[72] Salimpoor VN van den Bosch I Kovacevic N McIntosh AR Dagher A Zatorre RJ Interactions between the nucleus accumbens and auditory cortices predict music reward value. Science (2013) 340:216–9 10.1126/science.1231059 23580531

[73] Osuch EA Bluhm RL Williamson PC Theberge J Densmore M Neufeld RW Brain activation to favorite music in healthy controls and depressed patients. Neuroreport (2009) 20:1204–8 10.1097/WNR.0b013e32832f4da3 19617860

[74] Salamone JD Correa M The mysterious motivational functions of mesolimbic dopamine. Neuron (2012) 76:470–85 10.1016/j.neuron.2012.10.021 23141060 PMC4450094

[75] Howe MW Tierney PL Sandberg SG Phillips PE Graybiel AM Prolonged dopamine signalling in striatum signals proximity and value of distant rewards. Nature (2013) 500:575–9 10.1038/nature12475 23913271 PMC3927840

[76] Han DH Renshaw PF Sim ME Kim JI Arenella LS Lyoo IK The effect of internet video game play on clinical and extrapyramidal symptoms in patients with schizophrenia. Schizophr Res (2008) 103:338–40 10.1016/j.schres.2008.01.026 18304782

[77] Pompeu JE Arduini LA Botelho AR Fonseca MB Pompeu SM Torriani-Pasin C Feasibility, safety and outcomes of playing Kinect Adventures!™ for people with Parkinson’s disease: a pilot study. Physiotherapy (2014) 100:162–8 10.1016/j.physio.2013.10.003 24703891

[78] Mehndiratta P Sajatovic M Treatments for patients with comorbid epilepsy and depression: a systematic literature review. Epilepsy Behav (2013) 28:36–40 10.1016/j.yebeh.2013.03.029 23651914

[79] Schmidt D Sillanpaa M Evidence-based review on the natural history of the epilepsies. Curr Opin Neurol (2012) 25:159–63 10.1097/WCO.0b013e3283507e73 22274775

[80] Faught E Duh MS Weiner JR Guerin A Cunnington MC Nonadherence to antiepileptic drugs and increased mortality: findings from the RANSOM study. Neurology (2008) 71:1572–8 10.1212/01.wnl.0000319693.10338.b9 18565827

[81] Ettinger AB Manjunath R Candrilli SD Davis KL Prevalence and cost of nonadherence to antiepileptic drugs in elderly patients with epilepsy. Epilepsy Behav (2009) 14:324–9 10.1016/j.yebeh.2008.10.021 19028602

[82] Manjunath R Davis KL Candrilli SD Ettinger AB Association of antiepileptic drug nonadherence with risk of seizures in adults with epilepsy. Epilepsy Behav (2009) 14(2):372–8 10.1016/j.yebeh.2008.12.006 19126436

[83] Cardamone L Salzberg MR O’Brien TJ Jones NC Antidepressant therapy in epilepsy: can treating the comorbidities affect the underlying disorder? Br J Pharmacol (2013) 168:1531–54 10.1111/bph.12052 23146067 PMC3605864

[84] Lin LC Lee WT Wang CH Chen HL Wu HC Tsai CL Mozart K.448 acts as a potential add-on therapy in children with refractory epilepsy. Epilepsy Behav (2011) 20:490–3 10.1016/j.yebeh.2010.12.044 21292560

[85] Bozzi Y Borrelli E The role of dopamine signaling in epileptogenesis. Front Cell Neurosci (2013) 7:157 10.3389/fncel.2013.00157 24062645 PMC3774988

[86] Odano I Varrone A Savic I Ciumas C Karlsson P Jucaite A Quantitative PET analyses of regional [11C]PE2I binding to the dopamine transporter – application to juvenile myoclonic epilepsy. Neuroimage (2012) 59:3582–93 10.1016/j.neuroimage.2011.10.067 22056530

[87] Rocha L Alonso-Vanegas M Villeda-Hernandez J Mujica M Cisneros-Franco JM Lopez-Gomez M Dopamine abnormalities in the neocortex of patients with temporal lobe epilepsy. Neurobiol Dis (2012) 45:499–507 10.1016/j.nbd.2011.09.006 21964255

[88] Werhahn KJ Landvogt C Klimpe S Buchholz HG Yakushev I Siessmeier T Decreased dopamine D2/D3-receptor binding in temporal lobe epilepsy: an [18F]fallypride PET study. Epilepsia (2006) 47:1392–6 10.1111/j.1528-1167.2006.00561.x 16922886

[89] Allendorfer JB Heyse H Mendoza L Nelson EB Eliassen JC Storrs JM Physiologic and cortical response to acute psychosocial stress in left temporal lobe epilepsy – a pilot cross-sectional fMRI study. Epilepsy Behav (2014) 36C:115–23 10.1016/j.yebeh.2014.05.003 24907497

[90] Schmitt FC Voges J Heinze HJ Zaehle T Holtkamp M Kowski AB Safety and feasibility of nucleus accumbens stimulation in five patients with epilepsy. J Neurol (2014). 10.1007/s00415-014-7364-1 24801491 PMC4119256

[91] Raglio A Farina E Giovagnoli AR Can music therapy alleviate psychological, cognitive, and behavioral impairment in epilepsy? Epilepsy Behav (2014) 31:7–8 10.1016/j.yebeh.2013.10.008 24287099

[92] Modi AC Rausch JR Glauser TA Early pediatric antiepileptic drug nonadherence is related to lower long-term seizure freedom. Neurology (2014) 82:671–3 10.1212/WNL.0000000000000147 24463625 PMC3945661

[93] Tavakoli F Hoseini SE Mokhtari M Vahdati A Razmi N Vessal M Role of music in morphine rewarding effects in mice using conditioned place preference method. Neuro Endocrinol Lett (2012) 33:709–12 23391882

[94] Lin LC Juan CT Chang HW Chiang CT Wei RC Lee MW Mozart K.448 attenuates spontaneous absence seizure and related high-voltage rhythmic spike discharges in Long Evans rats. Epilepsy Res (2013) 104:234–40 10.1016/j.eplepsyres.2012.11.005 23395627

[95] Tasset I Quero I Garcia-Mayorgaz AD del Rio MC Tunez I Montilla P Changes caused by haloperidol are blocked by music in Wistar rat. J Physiol Biochem (2012) 68:175–9 10.1007/s13105-011-0129-8 22371013

[96] Lu Y Liu M Shi S Jiang H Yang L Liu X Effects of stress in early life on immune functions in rats with asthma and the effects of music therapy. J Asthma (2010) 47:526–31 10.3109/02770901003801964 20560827

[97] Rauscher FH Robinson KD Jens JJ Improved maze learning through early music exposure in rats. Neurol Res (1998) 20:427–32 9664590 10.1080/01616412.1998.11740543

[98] Rauscher FH Shaw GL Ky KN Listening to Mozart enhances spatial-temporal reasoning: towards a neurophysiological basis. Neurosci Lett (1995) 185:44–7 10.1016/0304-3940(94)11221-4 7731551

[99] Aoun P Jones T Shaw GL Bodner M Long-term enhancement of maze learning in mice via a generalized Mozart effect. Neurol Res (2005) 27:791–6 10.1179/016164105X63647 16354537

[100] Ramos A Lopez A Rodrigez A Meng T Abrams S The Legal Status of Video Games: Comparative Analysis in National Approaches. World Intellectual Property Organization (2013). p. 1–96 Available from: http://www.wipo.int/export/sites/www/copyright/en/activities/pdf/comparative_analysis_on_video_games.pdf

[101] DeFulio A Silverman K The use of incentives to reinforce medication adherence. Prev Med (2012) 55(Suppl):S86–94 10.1016/j.ypmed.2012.04.017 22580095 PMC3424340

[102] Brady TJ Murphy L Beauchesne D Bhalakia A Chervin D Daniels B Sorting Through the Evidence for the Arthritis Self-Management Program and the Chronic Disease Self-management Program. Atlanta: Center for Disease Control and Prevention (2011). p. 1–24

[103] White HS Loscher W Searching for the ideal antiepileptogenic agent in experimental models: single treatment versus combinatorial treatment strategies. Neurotherapeutics (2014) 11:373–84 10.1007/s13311-013-0250-1 24425186 PMC3996126

[104] Bucklen KW Abbott BM Promise and challenges with the use of mobile applications to support and improve patient care: an industry perspective. Clin Pharmacol Ther (2014) 95:469–71 10.1038/clpt.2014.34 24747232

[105] Robinson R What’s in a Game? Better Health Outcomes. PharmaVOICE (2014). Available from: http://www.pharmavoice.com/archives/article.esiml?id=2841

[106] Harrison C Patenting natural products just got harder. Nat Biotechnol (2014) 32:403–4 10.1038/nbt0514-403a 24811496

[107] Yetisen AK Martinez-Hurtado JL da Cruz Vasconcellos F Simsekler MC Akram MS Lowe CR The regulation of mobile medical applications. Lab Chip (2014) 14:833–40 10.1039/c3lc51235e 24425070

[108] Couto DS Perez-Breva L Saraiva P Cooney CL Lessons from innovation in drug-device combination products. Adv Drug Deliv Rev (2012) 64:69–77 10.1016/j.addr.2011.10.008 22200650

[109] Hupcey MA Ekins S Improving the drug selection and development process for combination devices. Drug Discov Today (2007) 12:844–52 10.1016/j.drudis.2007.07.020 17933686

[110] Food and Drug Administartion. Early Development Considerations for Innovative Combination Products, Guidance for Industry and FDA Staff (2006). Available from: http://www.fda.gov/regulatoryinformation/guidances/ucm126050.htm

[111] Food and Drug Administration. Office of Combination Products, Performance Report FY 2012 (2012). Available from: http://www.fda.gov/AboutFDA/ReportsManualsForms/Reports/PerformanceReports/CombinationProducts/ucm365878.htm

