# Supplementary material for: Combining Non-Pharmacological Treatments with Pharmacotherapies for Neurological Disorders: A Unique Interface of the Brain, Drug–Device, and Intellectual Property
Source: Front Neurol. 2014 Jul 14;5:126. doi: 10.3389/fneur.2014.00126 (PMC4095562; doi:10.3389/fneur.2014.00126)
Supplement: Supplementary file 1 [file Presentation_1.PDF]

Perspective

June 9th, 2014

Supplemental Information

Combining Non-pharmacological Treatments with Pharmacotherapies for Neurological Disorders: a Unique Interface of the Brain, Drug-Device and Intellectual Property

Grzegorz Bulaj

Table S1. Examples of mobile apps and games for neurological and psychiatric disorders.

App or Game	Clinical applications	References
SPARX [1]	depression	Merry SN et al, BMJ 2012, 344:e2598
SuperBetter	depression	www.SuperBetter.com
myCompass	depression, anxiety	Proudfoot et al, BMC Psychiatry 2013, 13:312
casual video games	depression	Russoniello CV et al, GHJ 2013, 2:341
Positive Technology	anxiety, stress	Gaggioli A et al, Stud Health Technol Inform 2014, 199: 25-29
Personal Zen [2]	anxiety	Dennis TA, O'Toole. Clin Psychol Sci March 2014
Evo	ADHD	www.akiliinteractive.com
NeuroRacer	dementia	Anguera JA et al, Nature 2013, 501:97
MINWii [3]	dementia	Boulay M et al, Technol Health Care 2011, 19:233
Reach+; Dodgewall	cerebral palsy	Ni LT et al, GHJ 2014, 3: 162-171
SnowWorld	burn pain	Hoffman HG et al, J Pain 2006, 7:843

[1] An example of a video game combining cognitive behavioral therapy and gamification; [2] This mobile app combines elements of attention-bias modification training and gamification; [3] This is an example of a music therapy game.

Table 2. Examples of brain fMRI studies on effects of music or games.

**Study**	**Main finding**	**Reference**
**GAMES**		
Re-Mission: comparing active and passive players	Specific activation of caudate nucleus and nucleus accumbens in active players	Cole SW et al, Plos One 2012, 7:e33909
BZFlag: comparing winning and losing	Self-acquired awards selectively activated orbitomedial prefrontal cortex	Katsyn J et al, Front Hum Neurosci 2013, 278:1
Gesture Therapy: rehabilitation-based game therapy for stroke patients	Activation of prefrontal cortex , cerebellar network	Orihuela-Espina F et al, Top Stroke Rehabil 2013, 20: 197
**MUSIC**		
Listening to music	Interactions between the nucleus accumbens and cortical network	Salimpoor VN et al, Science 2013, 340:216
Comparing pleasant and unpleasant music	Activation of the ventral stiatum and the caudate nucleus by favorite music	Montag C et al, Behav Brain Res 2011, 225:511
Comparing brain activation between depressed and healthy individuals	Increased activation of medial orbital frontal cortex, nucleus accumbens and ventral striatum in healthy volunteers	Osuch EA et al, Neuroreport 2009, 20:1204
